# Supplementary material for: Deconstructing body axis morphogenesis in zebrafish embryos using robot-assisted tissue micromanipulation
Source: Nat Commun. 2022 Dec 24;13:7934. doi: 10.1038/s41467-022-35632-4 (PMC9789989; doi:10.1038/s41467-022-35632-4)
Supplement: Supplementary file 3 — Description of Additional Supplementary Files [file 41467_2022_35632_MOESM3_ESM.pdf]

Description of Additional Supplementary Files

File Name: Supplementary Movie 1
Description: **Zebrafish embryo tail explant growth.** Bright-field time-lapse movie of a tail explant extending and elongating for 5 hours.

File Name: Supplementary Movie 2
Description: **Twitching of tail explant.** Bright-field movie of a twitching tail explant which completed somitogenesis.

File Name: Supplementary Movie 3
Description: **Buckling of the notochord of an embedded tail explant.** Example of a H2B-mCherry expressing explant growing in 2% LMPA indicating the buckled location's anterior progression every 1 hour for 8 hours.

File Name: Supplementary Movie 4
Description: **Balling up of a small posterior explant.** Bright-field time-lapse movie of a tail explant further dissected into anterior and posterior parts.

File Name: Supplementary Movie 5
Description: **Bilateral PSM ablation.** Elongation of the tail and the notochord of a H2B-mCherry expressing explant for 8 hours. Both sides of the posterior PSM are ablated.

File Name: Supplementary Movie 6
Description: **Her1-YFP signal of an explant after bilateral PSM ablation.** The expression of Her1-YFP signal in a tail explant. Signal is lost when the wave front passes the ablated region.

File Name: Supplementary Movie 7
Description: **Robot assisted microsurgery.** A live demonstration of cutting tail explants from zebrafish embryos using the robotic manipulation platform.

File Name: Supplementary Software 1
Description: STL files for the molds used to make the microsurgery and imaging chambers.
