# Supplementary material for: Deconstructing body axis morphogenesis in zebrafish embryos using robot-assisted tissue micromanipulation
Source: Nat Commun. 2022 Dec 24;13:7934. doi: 10.1038/s41467-022-35632-4 (PMC9789989; doi:10.1038/s41467-022-35632-4)
Supplement: Supplementary file 12 — Reporting Summary [file 41467_2022_35632_MOESM12_ESM.pdf]

nature portfolio

Corresponding author(s): Andrew C. Oates, Mahmut Selman Sakar

Last updated by author(s): Dec 5, 2022

Reporting Summary

Nature Portfolio wishes to improve the reproducibility of the work that we publish. This form provides structure for consistency and transparency in reporting. For further information on Nature Portfolio policies, see our Editorial Policies and the Editorial Policy Checklist.

Statistics

For all statistical analyses, confirm that the following items are present in the figure legend, table legend, main text, or Methods section.

n/a	Confirmed	
☐	☒	The exact sample size (n) for each experimental group/condition, given as a discrete number and unit of measurement
☐	☒	A statement on whether measurements were taken from distinct samples or whether the same sample was measured repeatedly
☐	☒	The statistical test(s) used AND whether they are one- or two-sided<br>*Only common tests should be described solely by name; describe more complex techniques in the Methods section.*
☐	☒	A description of all covariates tested
☒	☐	A description of any assumptions or corrections, such as tests of normality and adjustment for multiple comparisons
☐	☒	A full description of the statistical parameters including central tendency (e.g. means) or other basic estimates (e.g. regression coefficient) AND variation (e.g. standard deviation) or associated estimates of uncertainty (e.g. confidence intervals)
☐	☒	For null hypothesis testing, the test statistic (e.g. F, t, r) with confidence intervals, effect sizes, degrees of freedom and P value noted<br>*Give P values as exact values whenever suitable.*
☒	☐	For Bayesian analysis, information on the choice of priors and Markov chain Monte Carlo settings
☒	☐	For hierarchical and complex designs, identification of the appropriate level for tests and full reporting of outcomes
☒	☐	Estimates of effect sizes (e.g. Cohen's d, Pearson's r), indicating how they were calculated

Our web collection on statistics for biologists contains articles on many of the points above.

Software and code

Policy information about availability of computer code

Data collection	For image acquisition, we used Nikon Ti widefield microscope and Viventis LS1 live light-sheet microscope, and their respective acquisition software, NIS-Elements AR v5.02.01 and Viventis Microscope Control Software version 1.2.
Data analysis	BigDataViewer plugin (version 6.2.2) in FIJI (ImageJ 2.3.0/1.53f) was used to convert light-sheet images to xml/hdf5 format. Mastodon FIJI plugin (version 1.0.0-beta-20), Labkit plugin (version 0.3.0) and Paleontologist (version 0.4) were used to count cell nuclei in the time-lapse images. 3D views of explants were constructed using a commercial imaging software, Imaris Viewer x64 (version 9.7.0) Bitplane. Gaussian based stack focuser and LOI interpreter from Timelaps FIJI plugin (version 2.1.1) were used to quantify period of oscillations in the time-lapse images. All statistical tests were done with the built-in functions of commercial software OriginPro 2020b (Originlab).

For manuscripts utilizing custom algorithms or software that are central to the research but not yet described in published literature, software must be made available to editors and reviewers. We strongly encourage code deposition in a community repository (e.g. GitHub). See the Nature Portfolio guidelines for submitting code & software for further information.

Data

Policy information about availability of data

All manuscripts must include a data availability statement. This statement should provide the following information, where applicable:

- Accession codes, unique identifiers, or web links for publicly available datasets
- A description of any restrictions on data availability
- For clinical datasets or third party data, please ensure that the statement adheres to our policy

Data supporting the findings of this study are available within the paper and its Supplementary Information files. Raw image data are available upon request from the corresponding author.

Human research participants

Policy information about studies involving human research participants and Sex and Gender in Research.

Reporting on sex and gender	N/A
Population characteristics	N/A
Recruitment	N/A
Ethics oversight	N/A

Note that full information on the approval of the study protocol must also be provided in the manuscript.

Field-specific reporting

Please select the one below that is the best fit for your research. If you are not sure, read the appropriate sections before making your selection.

☒ Life sciences ☐ Behavioural & social sciences ☐ Ecological, evolutionary & environmental sciences

For a reference copy of the document with all sections, see nature.com/documents/nr-reporting-summary-flat.pdf

Life sciences study design

All studies must disclose on these points even when the disclosure is negative.

Sample size	In the experiments reported in the maintext, a total of n = 7 or more samples were used with minimum N = 3 independent runs. The numbers are determined based on the relevant literature (References 14, 15, 16, and 34 in the maintext) related to body axis elongation in vertebrates. In those articles, n was as small as 3, depending on the complexity and importance of the experiment.<br><br>In Supplementary Figures 4, 5, 8, and 12, n = 4 samples were reported and in Supplementary Figure 10 n = 6 samples were reported. The data shown in these figures support the arguments presented in the maintext by replicating the results using an alternative approach. They serve the purpose of validation and are not involved in the major conclusions of the study.
Data exclusions	Samples that went out of the field of view during light-sheet imaging and samples which were showing photo-bleaching were excluded for somitogenesis based period calculation.
Replication	Experiments performed on different days and with embryos from independent spawning were reliably reproduced. Number of repetition of the same experiment performed in different days is indicated in the figure legends with N. Experiments were performed at the same developmental stage, environmental temperature control at different stages of the experiments and using the same dedicated microscope for different experiments.
Randomization	For each experiment a population of samples coming from the same parents were selected based on viability and morphology. A further selection is done to select the samples that express the designated fluorescent protein. From this population of embryos, samples are randomly selected.
Blinding	During data collection the authors were blinded as embryos were selected randomly prior to measurement. During data analysis blinding was not possible since the phenotypes were quite distinct.

Reporting for specific materials, systems and methods

We require information from authors about some types of materials, experimental systems and methods used in many studies. Here, indicate whether each material, system or method listed is relevant to your study. If you are not sure if a list item applies to your research, read the appropriate section before selecting a response.

Materials & experimental systems

n/a	Involved in the study
☒	☐ Antibodies
☒	☐ Eukaryotic cell lines
☒	☐ Palaeontology and archaeology
☐	☒ Animals and other organisms
☒	☐ Clinical data
☒	☐ Dual use research of concern

Methods

n/a	Involved in the study
☒	☐ ChIP-seq
☒	☐ Flow cytometry
☒	☐ MRI-based neuroimaging

Animals and other research organisms

Policy information about studies involving animals; ARRIVE guidelines recommended for reporting animal research, and Sex and Gender in Research

Laboratory animals	Male and female adult zebrafish of her1+/-her7+/- and no tail (ntla, ntlb) mutants, and the following transgenic lines Her1::YFP, H2B::mCherry, utr::GFP, were maintained at the EPFL fish facility which has been accredited by the the Service de la Consommation et des Affaires Vétérinaires of the canton of Vaud – Switzerland (authorization number VD-H23). Embryos obtained from animals greater than 6 but less than 12 months old were used for experiments. Experiments were performed with 15 somite stage embryos.
Wild animals	There are no wild animals used in this study.
Reporting on sex	There is no sex based information in this study since at the developmental stages we performed the experiments the sex of the embryo is not determined yet.
Field-collected samples	There are no field-collected samples in this study.
Ethics oversight	The experiments in the paper were all carried out using embryos before 3 days of development, derived from freely mating adults therefore, they are covered by the general animal experimental license of EPFL, granted by the Service de la Consommation et des Affaires Vétérinaires of Canton Vaud-Switzerland (autorization number VD-H23).

Note that full information on the approval of the study protocol must also be provided in the manuscript.
